# Health-related quality of life and related factors in stroke survivors: Data from Korea National Health and Nutrition Examination Survey (KNHANES) 2008 to 2014

**DOI:** 10.1371/journal.pone.0195713

**Published:** 2018-04-10

**Authors:** SuYeon Kwon, Ji-Hong Park, Won-Seok Kim, Kyungdo Han, Yookyung Lee, Nam-Jong Paik

**Affiliations:** 1 Department of Rehabilitation Medicine, Seoul National University College of Medicine, Seoul National University Bundang Hospital, Seongnam-si, Gyeonggi-do, South Korea; 2 Department of Biostatistics, The Catholic University of Korea College of Medicine, Seoul, Korea; Hong Kong Polytechnic University, HONG KONG

## Abstract

As persons with stroke are surviving longer, monitoring and managing their quality of life is becoming important. We reviewed the Korea National Health and Nutrition Examination Survey (KNHANES) in order to evaluate the health-related quality of life (HRQoL) in stroke survivors as measured by the Euro Quality of Life-5D (EQ-5D), and to find out influencing factors. A total of 42,500 subjects were enrolled in the KNHANES, and 575 of them were persons with stroke. The EQ-5D index was lower in persons with stroke than those without stroke, when adjusted for age and sex (with stroke: 0.757±0.012, without stroke: 0.948±0.001, *p* < .0001). Dimension-specific influencing factors of HRQoL were observed in persons with stroke; mobility problems increased with old age; self-care problems increased with old age and depression; usual activity problems increased with old age, low income, absence of economic activity, and depression; pain/discomfort problems increased with low income. The EQ-5D index was lower in stroke survivors with older age, hypertension, diabetes mellitus, and lack of regular exercise. This is the first study to utilize nationally representative data of the Korean population to investigate the effect of stroke on HRQoL and explore the dimension-specific influencing factors. Further development of rehabilitative interventions for post-stroke depression, vocational rehabilitation, and tailored programs for encouraging physical activity may be needed to improve the HRQoL in Korean stroke survivors.

## Introduction

Stroke is a major health problem in Korea. It is one of the three most common causes of mortality, and its prevalence and related socioeconomic burdens are increasing.[1,2] Moreover, stroke causes functional impairments leading to dependency in activities of daily living (ADL), mood disorder, and social isolation, which consequently reduces the health related quality of life (HRQoL).[3–5] HRQoL is one of the major clinical outcomes in patients with stroke.[6,7] There have been many studies regarding HRQoL in persons with stroke, and their HRQoL was lower than those without stroke.[8] It is getting more important to monitor and improve HRQoL in stroke survivors because of improved survival following successful acute management.[9] Therefore, stroke rehabilitation has to be planned to improve the HRQoL, and identification of factors related with HRQoL is necessary to make an effective strategy.

Previous studies reported that age, depression, environmental factors and functional status such as Berg Balance Scale (BBS) or Fugl Meyer Assessment (FMA) are major influencing factors of HRQoL.[10–14] However, the estimates of HRQoL vary among studies,[15,16] and different cultural backgrounds also affect the degree of satisfaction in life.[13,17] Therefore, further research regarding HRQoL in diverse cultures would be meaningful in securing generality.

There was a recent report regarding the predictive factors of HRQoL using data from the cohort of stroke survivors in nine tertiary hospitals in Korea. The subjects were categorized into 5 groups according to the Euro Quality of Life-5D (EQ-5D) index measured at 6 months after stroke. The significant predictors of HRQoL measured by the EQ-5D were age, duration of hospitalization, and motor function at discharge.[18] However, this previous study lacks representativeness of general Korean stroke survivors, because only tertiary hospital based cohort data was used. In addition, dimension-specific analysis, which may provide results for more specific interventions to improve HRQoL, was not conducted.

Therefore, this study was designed primarily to investigate factors related with HRQoL in persons with stroke using nationally representative data, the Korea National Health and Nutrition Examination Survey (KNHANES) 2008 to 2014. In addition, factors related with each HRQoL dimension in EQ-5D, and differences in HRQoL between persons with stroke and those without stroke were investigated.

## Materials and methods

### Data source and participants

We used the KNHANES data from 2008 to 2014. The survey was conducted by the Korean Centers for Disease Control and Prevention (KCDCP). The KNHANES is a cross sectional and nationwide self-report on the health status including HRQoL.[19,20] The KNHANES has been conducted with an annual rolling sampling design including a complex, stratified, multistage probability-cluster survey of a representative Korean population sample aged 1 year and above.[21] A detailed description of the plan and operation of the survey is available on the KNHANES website (http://knhanes.cdc.go.kr/).

The participants were chosen by multistage stratification, and the survey included interviews, health behavior and nutrition survey, and a health examination study.[20] Among 42,500 participants who were 19 years or older, 575 participants who self-reported they had suffered from stroke or had seen a doctor for regular medical follow up due to stroke were enrolled as ‘persons with stroke’.

The Institutional Review Board at the Korea Centers for Disease Control and Prevention approved the protocol, and all participants signed informed consent forms. This study was approved by the Institutional Review Board of the Seoul National University Bundang Hospital. Informed consent was obtained from all participants when the KNHANES was originally conducted.

### Measures

The HRQoL was measured by the EQ-5D-3L questionnaire which includes 5 dimensions of mobility, self- care, usual activity, pain/discomfort, and anxiety/depression. Each dimension is answered by a three-point Likert scale; no problem, moderate problem, and severe problem.[22] The EQ-5D index, a single index of HRQoL ranging from 0 (dead) to 1 (full health), is then calculated based on responses to the 5-item questionnaire by using the population-based preference value of Korea.[23]

Sociodemographic factors such as age, sex, spouse status, education level, income level, and the presence of economic activity were collected. Clinical factors such as depression, regular exercise, obesity, current smoking status or alcohol consumption, hypertension, diabetes mellitus, and hypercholesterolemia were included.

Participants were stratified into three groups according to age (15–59, 60–69, and ≥70 years). Other sociodemographic and clinical variables were dichotomized into two groups. Spouse status was categorized as ‘Yes’ if living with a spouse, and ‘No’ if living alone. Educational level was categorized as lower than middle school graduation (≤9 years), and higher than high school graduation (≥10 years). The income level was categorized as low income (≤lowest quartile), and middle and high income. Economic activity was categorized as ‘Yes’ if currently employed, and ‘No’ if not employed or economically inactive. Regular exercise was defined as moderate or vigorous physical activity for at least 20 min performed at least three times per week. Obesity was defined as body mass index ≥25 kg/m^2^. Current smoking was defined as smoking at least one cigarette per day during the previous 12 months. Current drinking was defined as alcohol consumption in the past month. Depression was defined as feeling sad or despaired for at least 12 consecutive days during the previous one year. Hypertension was defined as blood pressure ≥140/90mmHg, or by use of antihypertensive medication. Diabetes mellitus was defined as fasting blood glucose ≥126mg/dL, or by use of hypoglycemic agents including insulin, or by self-reported physician’s diagnosis. Hypercholesterolemia was defined as serum total cholesterol ≥240mg/dL, or by use of dyslipidemia medication. Among the physiological variables, fasting blood glucose and total cholesterol were measured by using venous blood samples after a minimum fasting time of 8h. Blood pressure was measured using a mercury sphygmomanometer (Baumanometer; Baum, Copiague, NY, USA) in a seated position. Anthropometric measurements were performed with the subjects wearing light clothing, and body weight and height were measured to the nearest 0·1 kg and 0·1 cm, respectively.

### Statistical analysis

Statistical analysis was performed using SAS version 9.4, taking into account the complex sampling design of KNHANES. *P* values <0.05 were considered significant. We used stratification variables and sampling weights designated by the KCDCP based on the sample design of each survey year. Sociodemographic characteristics were compared between persons with and without stroke. Continuous variables were presented as mean (standard error of mean) and compared using the independent t-test. Categorical variables were presented as percentage (standard error of percentage) and compared using the Chi-square test.

HRQoL represented by the EQ-5D index in persons with stroke was compared to persons without stroke by using a general linear model, adjusting for age and sex.

A univariate analysis was conducted to investigate dimension-specific HRQoL in persons with stroke. Each 5 dimensions were categorized into ‘having problems’ and ‘having no problem’, and the two categorical variables were presented as percentage (standard error of percentage) values. Then, using the Chi-square test, the percentages of having problems in each dimension were compared among the three age groups and between dichotomized variables including spouse status, education level, income level, presence of economic activity, depression, regular exercise, obesity, current smoking or drinking, hypertension, diabetes mellitus, and hypercholesterolemia.

Multiple logistic regression was performed to investigate factors influencing each dimension of HRQoL. All 5 dimensions of HRQoL were categorized into 'having problems (including some problems and extreme problems)' and 'having no problem’, and odds ratios (ORs) and 95% confidence intervals (CIs) were estimated to present the risk of dimension-specific problem status caused by patients’ sociodemographic and health-related characteristics. The sociodemographic and health-related characteristics included in the multiple logistic regression analysis were the variables showing a possibly statistical association (*p* value < 0.25) with “having problems” in each five domain of EQ-5D in the univariate analysis. EQ-5D index was presented as mean (standard error of mean) and compared among the three age groups and between the dichotomized variables, using analysis of covariance (ANCOVA) with adjusting for possible confounders (age, sex, spouse, education level, income level, depression, regular exercise, hypertension, diabetes mellitus, and hypercholesterolemia). Bonferroni correction was applied for the post-hoc comparisons among three age groups, with the *p* value <0.017. Hosmer-Lemeshow's test was applied to test the goodness-of-fit for the logistic regression model, and *p* values > 0.05 indicated the regression model fit.

## Results

### Characteristics of subjects

The sociodemographic and clinical characteristics are shown in Table 1. Among 42,500 subjects in our sample, 575 were persons with stroke. The mean age of the persons with stroke was 64.8±0.6 years, and that of persons without stroke was 45.1±0.2 years. Persons with stroke were predominantly male, and most of them were living without their spouse. Persons with stroke had less education, lower income level and less economic activity compared to those without stroke. The mean time since diagnosis of stroke was 8.4±0.4 years. Persons with stroke showed significantly higher prevalence of hypertension and diabetes mellitus compared to those without stroke.

**Table 1:** Demographic and clinical characteristics of persons with and without history of stroke.

	Persons without history of stroke (n = 41,925)	Persons with history of stroke (n = 575)	*P value*
**Age**			<0.001
19–59	80.4 (0.32)	30.4 (2.5)	
60–69	10.5 (0.19)	32.1 (2.2)	
≥70	9 (0.2)	37.5 (2.3)	
**Sex**			0.001
Male	49.1 (0.25)	57.4 (2.3)	
Female	50.9 (0.25)	42.6 (2.3)	
**Presence of spouse**			<0.001
No	78 (0.38)	96.7 (0.96)	
Yes	21.9 (0.38)	3.3 (0.96)	
**Educational level**			<0.001
≤ 9 years	28.2 (0.42)	71.3 (2.32)	
≥ 10 years	71.8 (0.42)	28.7 (2.32)	
**Income level**			<0.001
Middle and high income	84.4 (0.34)	50.8 (2.5)	
Low income	15.6 (0.34)	49.2 (2.5)	
**Economic activity**			0.007
No	36.7 (0.34)	71.8 (2.33)	
Yes	63.3 (0.34)	28.2 (2.33)	
**Time since stroke onset(years)**	-	8.4 (0.4)	
**Current smoking**			<0.001
No	76 (0.28)	84.7 (1.78)	
Yes	24 (0.28)	15.3 (1.78)	
**Current drinking**			<0.001
No	41.4 (0.33)	64.3 (2.47)	
Yes	58.6 (0.33)	35.7 (2.47)	
**Obesity**			<0.001
No	68.3 (0.3)	62.2 (2.34)	
Yes	31.7 (0.3)	37.8 (2.34)	
**Regular exercise**			0.451
No	58.8 (0.34)	60.7 (2.48)	
Yes	41.2 (0.34)	39.3 (2.48)	
**Depression**			<0.001
No	86.8 (0.23)	78.3 (2.09)	
Yes	13.2 (0.23)	21.7 (2.09)	
**Hypertension**			<0.001
No	74.3 (0.32)	29.9 (2.31)	
Yes	25.7 (0.32)	70.1 (2.31)	
**Diabetes mellitus**			<0.001
No	91.6 (0.17)	67.9 (2.59)	
Yes	8.4 (0.17)	32.1 (2.59)	
**Hypercholesterolemia**			<0.001
No	88.3 (0.2)	72.4 (2.41)	
Yes	11.7 (0.2)	27.6 (2.41)	

Values in ‘time since stroke onset (years) is mean (standard error of mean), and the other values are percentages (standard error of percentages).

### Comparison of HRQoL between persons with and without stroke

The EQ-5D index was compared between persons with stroke and those without stroke, adjusting for age and sex. The EQ-5D index was lower in persons with stroke (with stroke: 0.757±0.012, without stroke: 0.948±0.001, *p*<0.0001) suggesting significant impairment of HRQoL in the stroke population, as generally expected.

**Fig 1:**
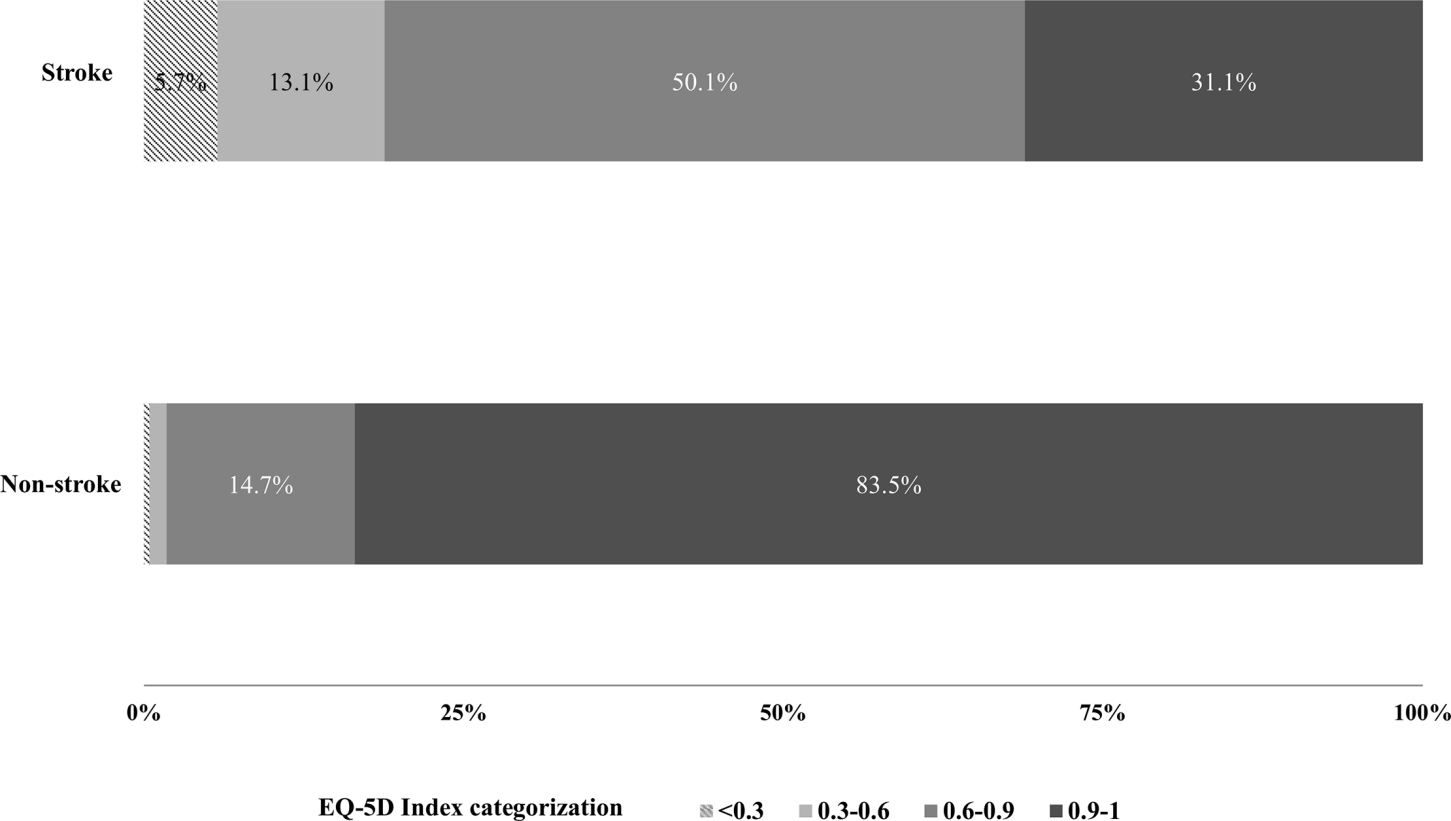
Distribution of HRQoL in stroke and non-stroke subjects. The four categorization of EQ-5D index was used; 0.9–1 for satisfied; 0.6–0.9 for little dissatisfied; 0.3–0.6 for moderately dissatisfied; <0.3 for severely dissatisfied.

The distribution of HRQoL was further analyzed. The four categorization of EQ-5D index was used; 0.9–1 for satisfied; 0.6–0.9 for little dissatisfied; 0.3–0.6 for moderately dissatisfied; <0.3 for severely dissatisfied.[18] In the stroke population, the dissatisfied status was considerably higher than the non-stroke population. Little dissatisfied status accounted for 50.1% of stroke population, moderately dissatisfied 13.1% (vs. 1.3% in non-stroke), and severely dissatisfied 5.7% (vs. 0.4% in non-stroke) respectively (Fig 1).

### Problem status and influencing factors of HRQoL in persons with stroke

In the univariate analysis, old age, male, absence of spouse, low educational level, low income, absence of economic activity, lack of regular exercise, depression, and diabetes mellitus were related with higher percentage of ‘having problems’ in one of five dimensions of HRQoL (Table 2).

**Table 2:** Prevalence of having problems in each EQ-5D dimension among persons with history of stroke (n = 575).

	Mobility	Self-care	Usual activity	Pain/Discomfort	Anxiety/Depression
**Age**					
19–59	41.1 (5.3)	22.9 (4.7)	37.6 (5.4)	46.1 (5.2)	22.9 (4.4)
60–69	61.9 (4.3)	37.9 (4)	54 (4.3)	56.4 (4.1)	31 (3.5)
≥70	71.7 (3.5)	41.4 (3.6)	65.8 (3.5)	65.1 (3.7)	29.6 (3.2)
*P value*	<0.001*	0.005*	<0.001*	0.009*	0.300
**Sex**					
Male	57.5 (3.5)	34.1 (3.3)	51.6 (3.4)	52.6 (3.2)	22.6 (2.8)
Female	61.6 (3.6)	35.5 (3.5)	55.9 (3.7)	61.8 (3.6)	35.3 (3.4)
*P value*	0.399	0.762	0.372	0.045*	0.005*
**Spouse**					
No	66.3 (5)	43.1 (5)	59 (5.1)	63.7 (5)	35.2 (4.4)
Yes	56.8 (2.8)	31 (2.5)	51.7 (2.8)	54 (2.8)	24.2 (2.3)
*P value*	0.104	0.019*	0.200	0.101	0.024*
**Education**					
≤9 years	64.4 (2.9)	35.7 (2.9)	58.5 (3)	59.7 (2.8)	30.2 (2.6)
≥10 years	46.7 (4.9)	31.9 (4.7)	40.4 (4.5)	48.3 (4.8)	22.3 (4.3)
*P value*	0.002*	0.481	0.001*	0.033*	0.149
**Income**					
Middle and high income	50 (3.6)	25.6 (3)	39.8 (3.3)	46.6 (3.4)	20.4 (2.6)
Low income	69.3 (3.2)	44.4 (3.7)	67 (3.3)	67.6 (3.3)	36.1 (3.4)
*P value*	<0.001*	<0.001*	<0.001*	<0.001*	<0.001*
**Economic activity**					
No	66.1 (2.7)	40.6 (2.9)	61.3 (2.9)	62.8 (2.8)	30.4 (2.5)
Yes	41.6 (5)	19.2 (4)	32.5 (4.7)	40.6 (5)	21.4 (3.7)
*P value*	<0.001*	<0.001*	<0.001*	<0.001*	0.052
**Current smoking**					
No	58.5 (2.8)	33.3 (2.6)	52.2 (2.8)	55 (2.8)	28 (2.2)
Yes	64.3 (6.1)	40.1 (6.1)	58 (6.3)	61.3 (6.3)	26.9 (5.6)
*P value*	0.390	0.271	0.395	0.362	0.863
**Current drinking**					
No	60.4 (3.2)	36.4 (3.1)	52.9 (3.1)	58.7 (3.1)	28.6 (2.6)
Yes	57.3 (4.3)	30.9 (4.2)	53.6 (4.4)	51.7 (4.1)	26.1 (3.6)
*P value*	0.555	0.285	0.893	0.158	0.565
**Obesity**					
No	57.5 (3.1)	34 (3)	54.1 (3.2)	54.8 (3.1)	28.1 (2.9)
Yes	60.9 (3.9)	34.2 (4)	51.9 (4.1)	58.1 (4.2)	28.1 (3.5)
*P value*	0.462	0.967	0.668	0.517	0.993
**Regular exercise**					
No	65.4 (3.1)	38.4 (3.3)	57.7 (3.2)	58.8 (3.2)	30.4 (3.1)
Yes	49.7 (4)	28 (3.4)	46.5 (4.1)	52.1 (4.2)	24.6 (3.1)
*P value*	0.002*	0.023*	0.026*	0.213	0.206
**Depression**					
No	57.2 (3)	29.8 (2.8)	47.9 (3.1)	53.5 (3.1)	18.6 (2.2)
Yes	69.2 (5.4)	46.6 (5.5)	66.7 (5.2)	67.2 (5.4)	51.1 (5.4)
*P value*	0.059	0.004*	0.002*	0.030*	<0.001*
**Hypertension**					
No	50.9 (4.6)	28.8 (4)	53.3 (4.7)	49.6 (4.8)	30.1 (4.4)
Yes	62.8 (3.1)	37.2 (3)	53.5 (3)	59.5 (2.8)	27.1 (2.3)
*P value*	0.032*	0.100	0.975	0.067	0.541
**Diabetes mellitus**					
No	51.8 (3.3)	27.8 (3)	44.5 (3.4)	52.5 (3.4)	27.2 (2.9)
Yes	67.2 (4.6)	41.5 (4.7)	61.8 (4.5)	60.9 (4.2)	27.4 (4.1)
*P value*	0.006*	0.009*	0.001*	0.128	0.980
**Hypercholesterolemia**					
No	56.9 (3.1)	28 (2.8)	49.1 (3.2)	54.2 (3)	25.6 (2.8)
Yes	56.3 (5.6)	42.4 (5.6)	52.3 (5.4)	57.8 (5.5)	31.8 (5)
*P value*	0.920	0.013*	0.591	0.560	0.269

Values are presented as percentage (standard error of percentage). *P* values are calculated by chi-square test.

*p<0.05.

The multivariate analysis revealed dimension-specific influencing factors of HRQoL in stroke survivors (Table 3). Mobility problems were 2.91 times higher in persons with stroke in their sixties and 4.75 times higher in 70 years or older, compared to those younger than sixty years. By contrast, mobility problems were 0.59 times lower in person with stroke who performed regular exercise, compared to those who did not. Self-care problems were 2.88 times higher in persons with stroke in their sixties and 2.59 times higher in 70 years or older, compared to those younger than sixty years. Self-care problems were also 2.13 times higher in persons with stroke who felt depressed. Usual activity problems were 2.42 times higher in persons with stroke 70 years or older, compared to those younger than sixty years. Usual activity problems were 2.60 times higher in person with stroke who had a low income compare to those with middle and high income; 2.13 times higher in those with inactive economic status; 2.33 times higher in those with depression. Pain/discomfort problems were 2.26 times higher in those who had a low income compare to those with middle and high income.

**Table 3:** Risk factors for having problems in each EQ-5D dimension among persons with history of stroke (n = 575): A multiple logistic regression analysis.

Variables	Mobility	Self-care	Usual activity	Pain/Discomfort	Anxiety/Depression
**Age**					
19–59	Reference	Reference	Reference	Reference	Reference
60–69	2.91 (1.54–5.53)‡	2.88 (1.35–6.16)*	1.77 (0.93–3.37)	1.63 (0.85–3.10)	2.15 (0.99–4.69)
≥70	4.75 (2.33–9.67)‡	2.59 (1.26–5.35)*	2.42 (1.27–4.60)*	1.98 (1.00–3.90)	1.73 (0.78–3.84)
**Female**	-	-	-	-	1.01 (0.58–1.74)
**No spouse**	1.16 (0.62,2.18)	-	-	-	-
**Low educational level** (≤9-year educational period)	1.22 (0.67–2.24)	1.71 (0.88–3.32)	-	-	-
**Low income** (≤lowest quartile)	1.62 (0.93–2.83)	1.68 (0.94–3.00)	2.60 (1.53–4.42)†	2.26 (1.34–3.82)†	1.76 (0.89–3.46)
**No economic activity**	1.54 (0.86–2.76)	1.86 (0.90–3.84)	2.13 (1.15–3.96)*	1.30 (0.72–2.34)	0.93 (0.47–1.86)
**Regular exercise**	0.59 (0.35–0.97)*	0.62 (0.36–1.06)	0.68 (0.42–1.11)	0.79 (0.46–1.33)	0.94 (0.54–1.64)
**Monthly drinking**	-	-	-	0.91 (0.53–1.55)	-
**Obesity**	1.29 (0.75–2.24)	1.50 (0.87–2.58)	1.18 (0.71–1.96)	1.19 (0.71–2.02)	1.41 (0.78–2.55)
**Depression**	1.31 (0.73–2.36)	2.13 (1.19–3.81)*	2.33 (1.25–4.34)*	1.14 (0.58–2.22)	3.25 (1.63–6.46)†
***p-*value of chi-square statistics**	0.92**	0.55**	0.16**	0.20**	0.22**

Values are presented as odds ratio (95% confidence interval).

*p<0.05

†*p*<0.005

‡*p*<0.0001

Hosmer-Lemeshow's test (*p-*value of chi-square statistics) was performed to test the goodness-of-fit for the logistic regression model.

**p> 0.05.

**Fig 2:**
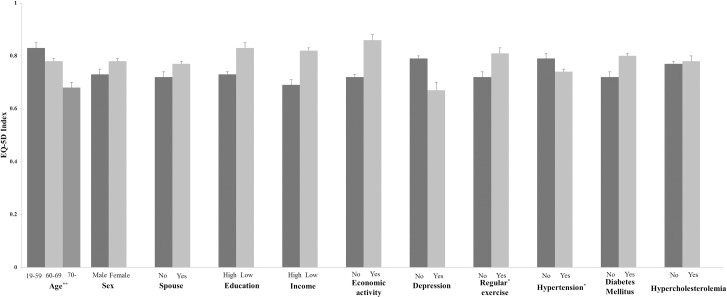
Influencing factors of overall HRQoL in stroke patients. The EQ-5D index was lower in persons with stroke with old age (19–59: 0.89±0.02, 60–69: 0.85±0.03, ≥70: 0.78±0.03, *p*<0.005). The EQ-5D index was also lower in persons with stroke with hypertension (with hypertension: 0.74±0.01, without hypertension: 0.79±0.02, *p*<0.05), and lack of regular exercise (without regular exercise: 0.82±0.03, with regular exercise: 0.86±0.02, *p*<0.05). The confounding effects among the sociodemographic and health-related characteristics were adjusted for. The values are mean±standard error of mean. *p<0.05 and **p<0.005.

Lower mean values of EQ-5D index were observed in persons with stroke with old age, hypertension, and lack of regular exercise, when the confounding effects among the sociodemographic and health-related characteristics were adjusted for (Fig 2). In the post-hoc analysis among three age groups, only the difference between 19≤age≤59 and age≥70 was statistically significant (*p* = 0.0005).

## Discussion

In this study, the EQ-5D index in persons with stroke was lower than those without stroke as expected, and about 50% of all stroke survivors showed ‘little dissatisfied HRQoL’. People with ‘satisfied’ HRQoL comprised only 31% of stroke survivors. In contrast, 84% of persons with stroke were ‘satisfied’ (Fig 1). This result is consistent with previous studies [3–5,8] and suggests the need for further research to find out the possible modifiable influencing factors and interventions to reduce the gap in the HRQoL in Korean stroke survivors.

Stroke survivors with older age, no regular exercise showed lower HRQoL (Fig 2). The average age of stroke survivors in this study was 64.8 years, similar to previous studies. [13,24] The role of age on HRQoL has not been conclusive in literature regarding stroke survivors. Some studies showed that age had a strong influence on quality of life,[11,25] while others found that there was no negative influence of age in patients with stroke.[24,26] According to our findings, age was closely related to mobility, self-care and usual activity in patients with stroke (Table 3). Age has been known as a strong predictor of functional recovery after stroke,[27,28] as comorbidities increase with aging. So it is reasonable to infer that age may affect quality of life in patients with stroke. Previous studies have shown that physical activity is associated with better post-stroke HRQoL.[29,30] In this study, no regular exercise was also associated with worse HRQoL and more problems in mobility. Although the degree of motor impairment was not considered in this study, efforts to increase physical activity among Korean stroke survivors could improve the HRQoL, especially in the physical domain. Tailored programs according to the level of physical performance in stroke survivors (e.g. encouraging leisure activities for the mild active stroke survivors, community-based rehabilitation with assistance of physical therapists or devices for the severe stroke) have to be considered.[31,32]

In the dimension-specific analysis, there were distinct increases in the chance of having problems in usual activity and pain/discomfort in patients with stroke who had lower income or no economic activity (Table 3). The association between lower socioeconomic status and increased recurrence, mortality, and severity in stroke has been reported.[33] This may imply that patients with lower income could not afford sufficient treatment and rehabilitation after stroke and as a result, have recovered incompletely.[34] The insufficient financial support also restricts medical care and participation in activities, which in turn aggravates the lower quality of life in stroke survivors.[35] In addition, because stroke survivors showed low chances of returning to their previous jobs and it depends on their stroke severity,[36] vocational rehabilitation program should also be considered as one of the post-stroke rehabilitation program.

Depression was shown to be correlated with dimensions such as self-care and usual activity (Table 3). Post-stroke depression is common,[37] and 21.7% of stroke survivors had self-reported depressive mood in our study. Post-stroke depression is associated with worse recovery and outcomes in multiple functional domains such as activity limitations and participation restrictions,[38–40] which leads to worse HRQoL in stroke survivors.[41] Therefore, comprehensive medical attention including treatment of depression should be provided in post-stroke rehabilitation, and depression screening and community-based interventions with support from other family members or care-givers has to be considered in chronic stroke survivors.

This study has several limitations. First, the presence of stroke was decided by using questions during interview and was not confirmed by medical record or brain imaging review. Secondly, severity and various functional impairments (e.g. cognitive impairment, aphasia, hemispatial neglect) of stroke were not considered for analysis. Thirdly, causal relationship cannot be clarified due to the cross-sectional study design. Despite these limitations, the strength of this study is that we used nationally representative data of stroke patients in Korea, which is more advantageous in terms of external validity than studies based on regional or hospital data, and we reported the quality of life in 5 different dimensions with related factors.

## Conclusion

This is the first study to demonstrate the effect of stroke on HRQoL and explore the dimension-specific influencing factors using the nationally representative data of the Korean population. Stroke survivors show worse HRQoL than persons without stroke. Old age, less physical activity, low income, and depression is associated with worse general HRQoL or more problems in specific domains among stroke survivors. Further development of rehabilitative interventions for post-stroke depression, vocational rehabilitation, and tailored program for encouraging physical activity may be needed to improve the HRQoL in Korean stroke survivors.
